# Supplementary material for: R-spondin 3 deletion induces Erk phosphorylation to enhance Wnt signaling and promote bone formation in the appendicular skeleton
Source: eLife. 2022 Nov 2;11:e84171. doi: 10.7554/eLife.84171 (PMC9681208; doi:10.7554/eLife.84171)
Supplement: Figure 5—source data 1. — Representative image active β-catenin by Western analysis in BMSC isolated from WT and Rspo3+/- mice (n=7). [file elife-84171-fig5-data1.zip › Figure 5b-source data 1/Figure 5b-source data 1-uncropped labelled blots.docx]

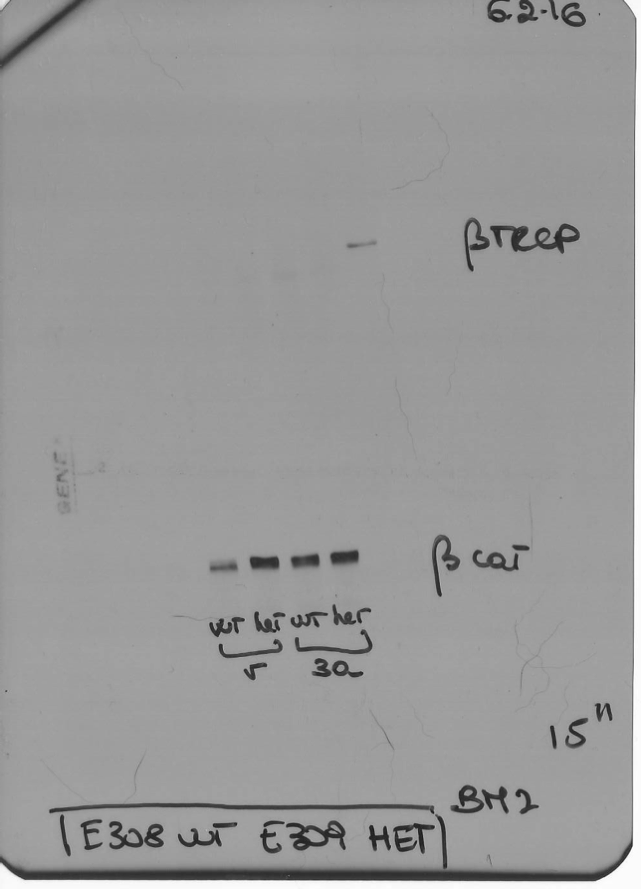


Figure 5b. Source data 1. Representative images of uncropped blots of active β-catenin in BMSC isolated from *wt* and *Rspo3^+/-^* mice
