# Supplementary figures and images for: R-spondin 3 deletion induces Erk phosphorylation to enhance Wnt signaling and promote bone formation in the appendicular skeleton
Source: eLife. 2022 Nov 2;11:e84171. doi: 10.7554/eLife.84171 (PMC9681208; doi:10.7554/eLife.84171)

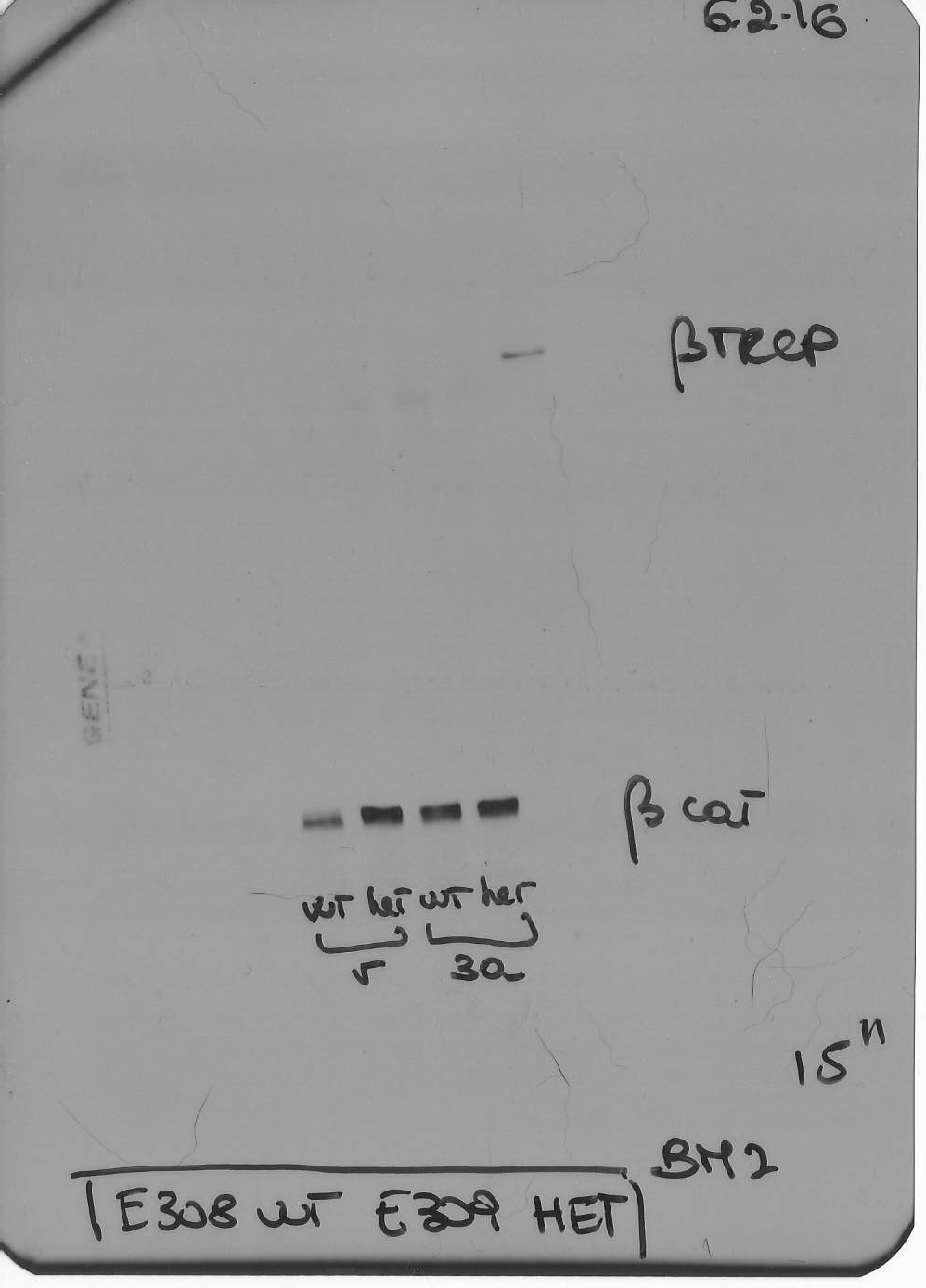

Supplement: Figure 5—source data 1. — Representative image active β-catenin by Western analysis in BMSC isolated from WT and Rspo3+/- mice (n=7). [file elife-84171-fig5-data1.zip › Figure 5b-source data 1/Figure 5b-source data 1- bcatenin.tif]

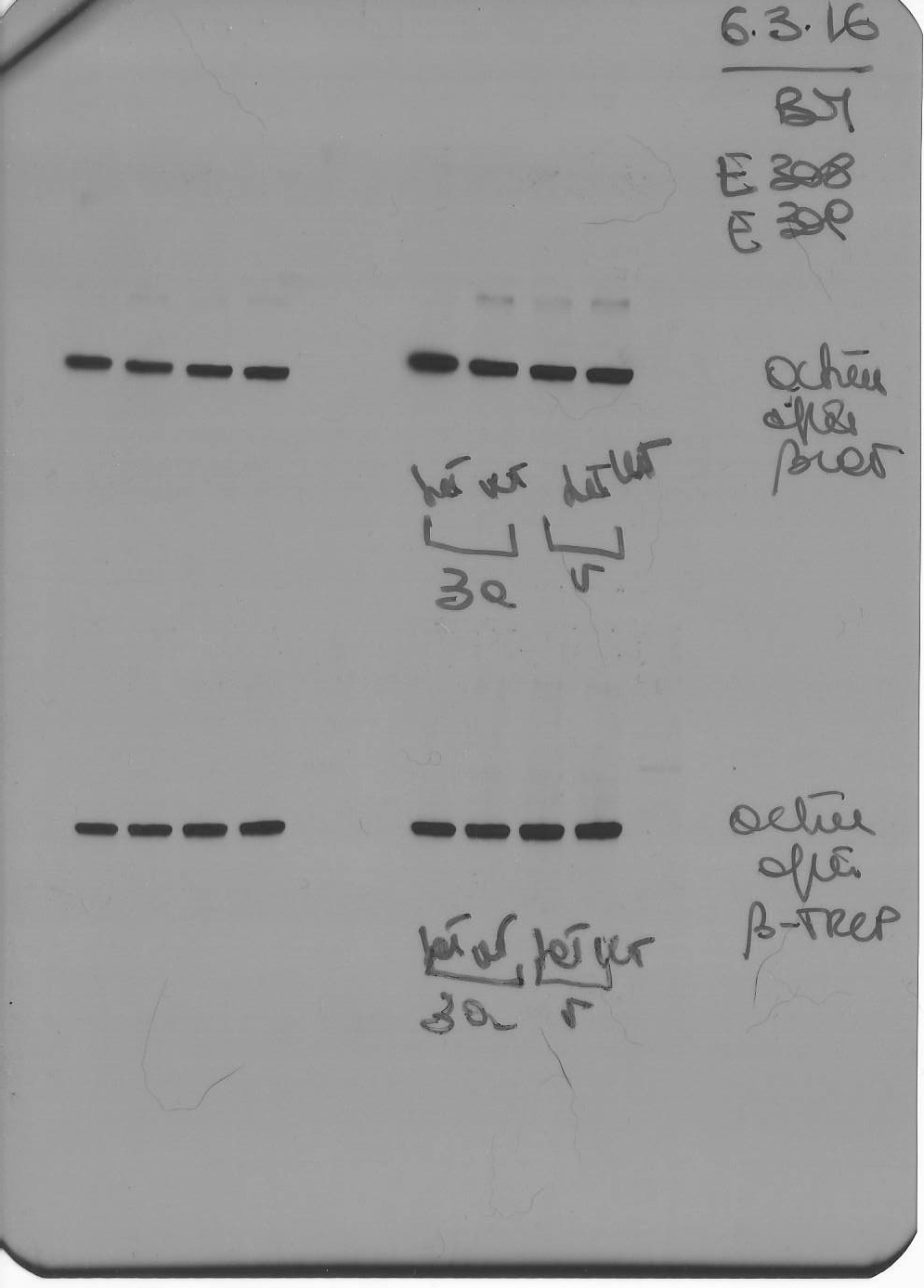

Supplement: Figure 5—source data 2. — Representative image of actin by Western analysis in BMSC isolated from WT and Rspo3+/- mice (n=7). [file elife-84171-fig5-data2.zip › Figure 5b-Source data-2/Figure 5b-source data 2-actin .tif]

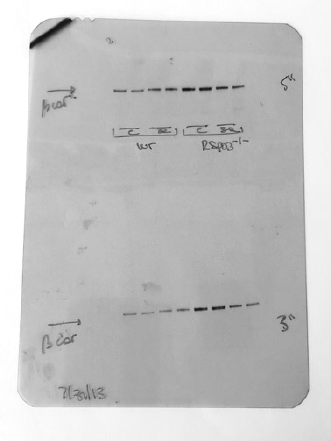

Supplement: Figure 6—source data 1. — Representative image of active β−catenin by Western analysis in WT and Rspo-/- MEFs treated w/wo Wnt3a (n=7). [file elife-84171-fig6-data1.zip › Figure 6b-source data 1/Figure 6b-source data 1-bcatenin.tif]

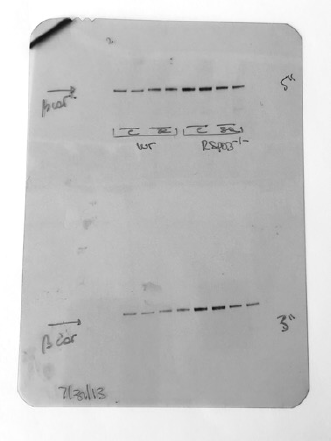


Representative uncropped labelled blot of active β−catenin in *wt* and *Rspo-/-*MEFs treated w/wo Wnt3a

Supplement: Figure 6—source data 1. — Representative image of active β−catenin by Western analysis in WT and Rspo-/- MEFs treated w/wo Wnt3a (n=7). [file elife-84171-fig6-data1.zip › Figure 6b-source data 1/Figure 6b-source data 1_uncropped labelled blot.docx]

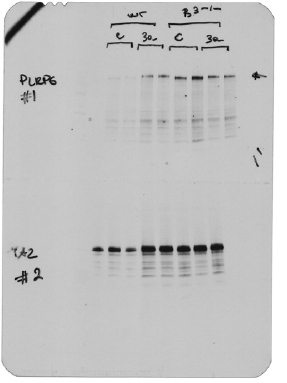

Supplement: Figure 6—source data 2. — Representative image of pLrp6 by Western analysis in WT and Rspo-/- MEFs treated w/wo Wnt3a (n=7). [file elife-84171-fig6-data2.zip › Figure 6b-source data 2/Figure 6b-source data 2-PLRP6.tif]

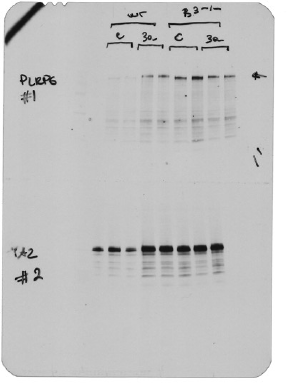


Representative uncropped labelled blot of pLrp6 in *wt* and *Rspo-/-*MEFs treated w/wo Wnt3a

Supplement: Figure 6—source data 2. — Representative image of pLrp6 by Western analysis in WT and Rspo-/- MEFs treated w/wo Wnt3a (n=7). [file elife-84171-fig6-data2.zip › Figure 6b-source data 2/Figure 6b-source data 2_uncropped labelled blot.docx]

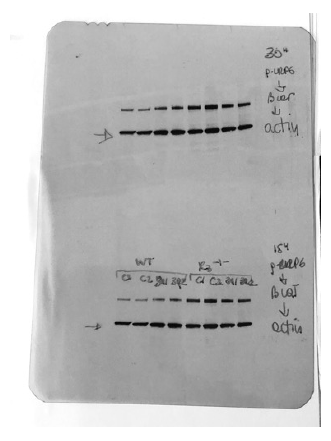


Representative uncropped labelled blot of actin in *wt* and *Rspo-/-*MEFs treated w/wo Wnt3a

Supplement: Figure 6—source data 3. — Representative image of actin by Western analysis in WT and Rspo-/- MEFs treated w/wo Wnt3a (n=7). [file elife-84171-fig6-data3.zip › Figure 6b-source data 3/Figure 6b-source data 3_uncropped labelled blot.docx]

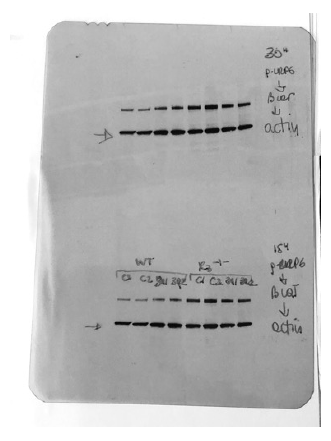

Supplement: Figure 6—source data 3. — Representative image of actin by Western analysis in WT and Rspo-/- MEFs treated w/wo Wnt3a (n=7). [file elife-84171-fig6-data3.zip › Figure 6b-source data 3/Figure 6b-source data 3-actin.tif]

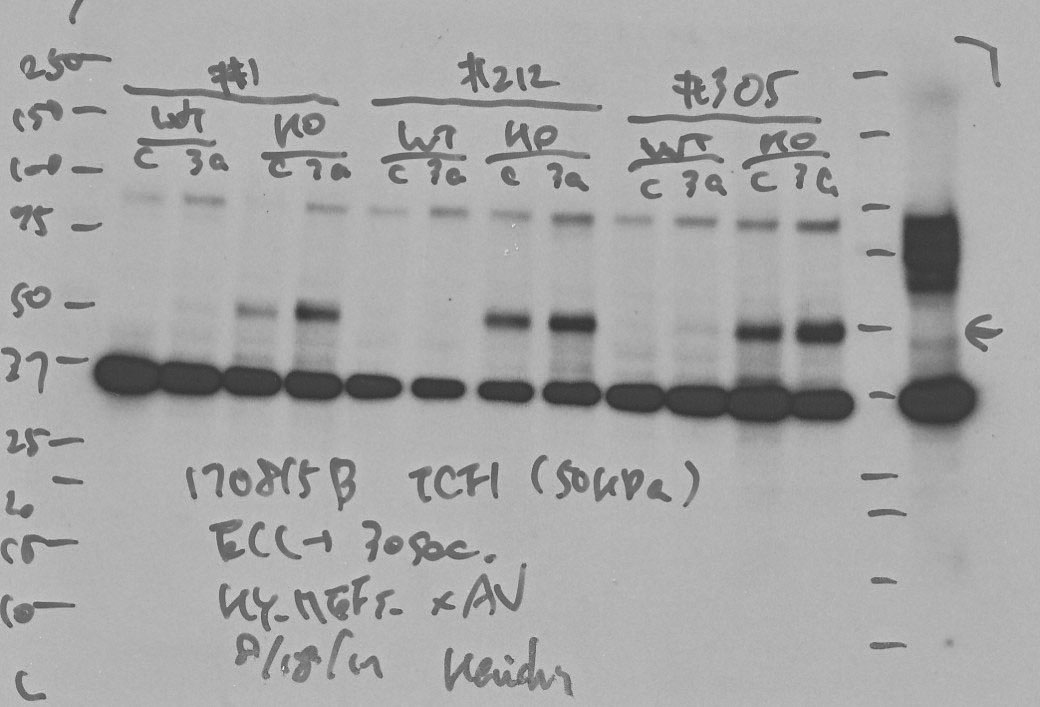

Supplement: Figure 6—source data 4. — Representative image of Tcf1 by Western analysis in WT and Rspo-/- MEFs treated w/wo Wnt3a (n=3). [file elife-84171-fig6-data4.zip › Figure 6b-source data 4/Figure 6b-source data 4-Tcf1.tif]

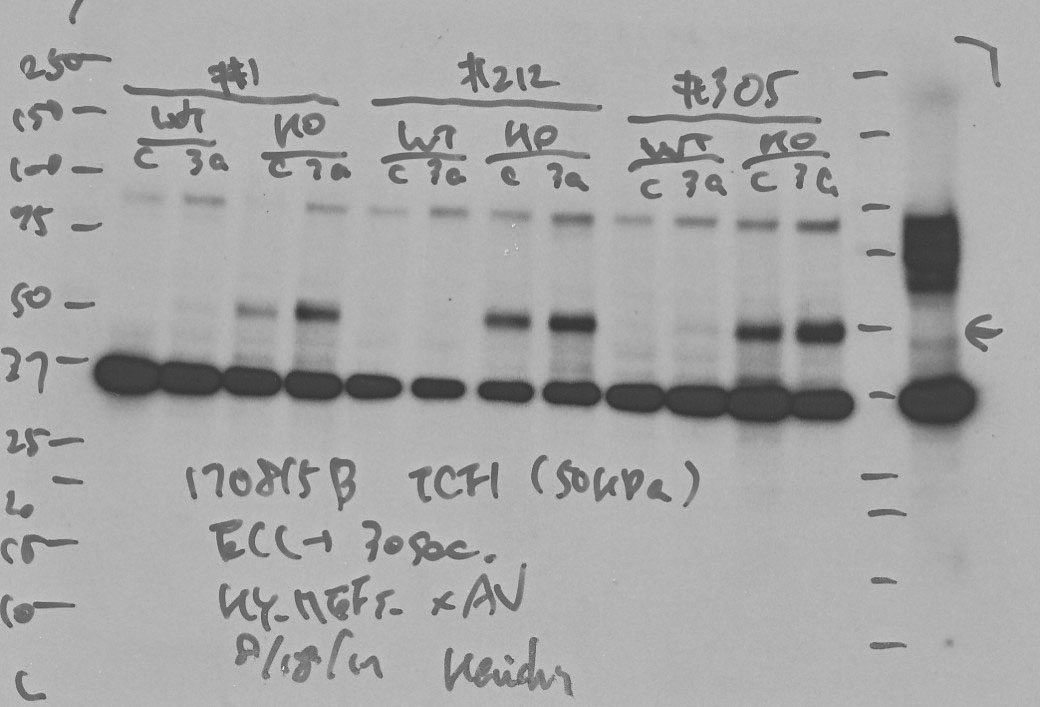


Representative uncropped labelled blot of Tcf1 in *wt* and *Rspo-/-*MEFs treated w/wo Wnt3a

Supplement: Figure 6—source data 4. — Representative image of Tcf1 by Western analysis in WT and Rspo-/- MEFs treated w/wo Wnt3a (n=3). [file elife-84171-fig6-data4.zip › Figure 6b-source data 4/Figure 6b-source data 4_uncropped labelled blot.docx]

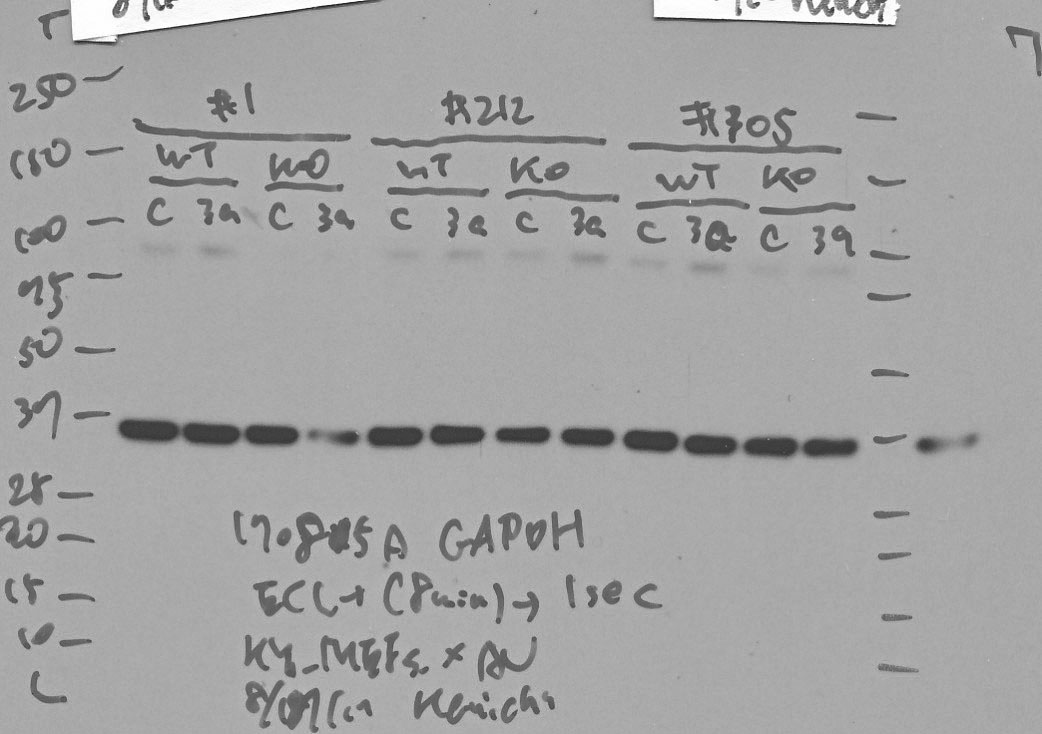


Representative uncropped labelled blot of Gapdh in *wt* and *Rspo-/-*MEFs treated w/wo Wnt3a

Supplement: Figure 6—source data 5. — Representative image of Gapdh by Western analysis in WT and Rspo-/- MEFs treated w/wo Wnt3a (n=3). [file elife-84171-fig6-data5.zip › Figure 6b-source data 5/Figure 6b-source data 5_uncropped labelled blot.docx]

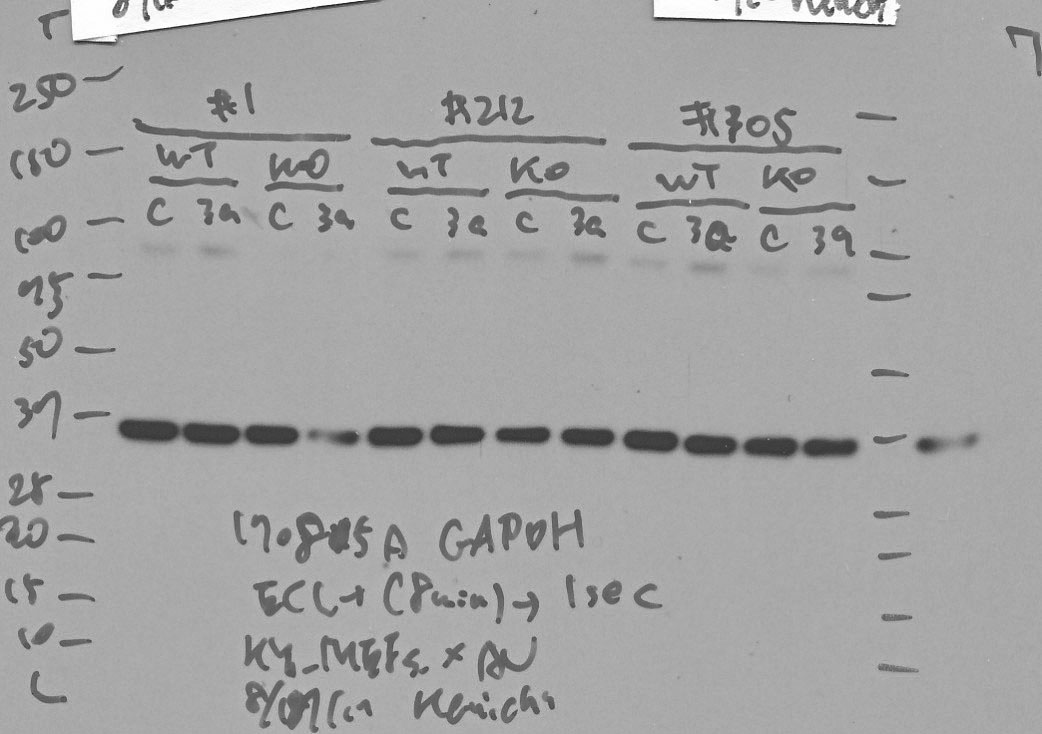

Supplement: Figure 6—source data 5. — Representative image of Gapdh by Western analysis in WT and Rspo-/- MEFs treated w/wo Wnt3a (n=3). [file elife-84171-fig6-data5.zip › Figure 6b-source data 5/Figure 6b-source data 5-GAPDH.tif]

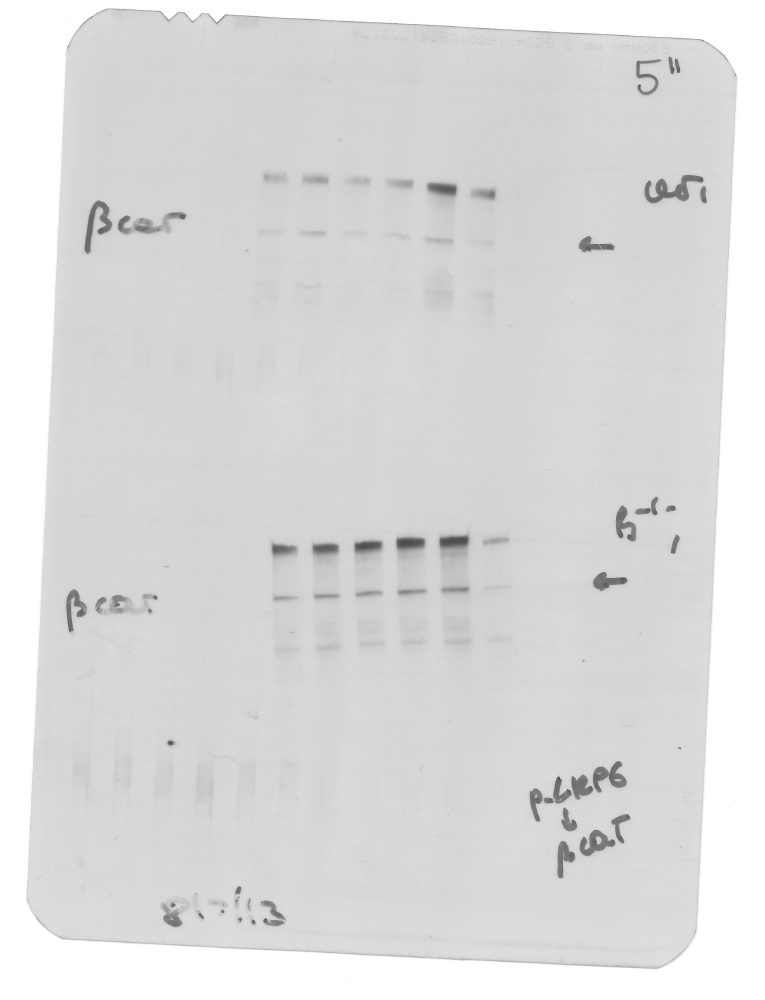

Supplement: Figure 7—source data 1. — Representative image of active β-catenin and pLrp6 by Western analysis in WT and Rspo3-/- MEFs treated w/wo Wnt3a and increasing doses of Dkk1 (n=3). [file elife-84171-fig7-data1.zip › Figure 7c-source data 1-2/Figure 7c-source data 1-2-Bcatenin_PLRP6.tif]

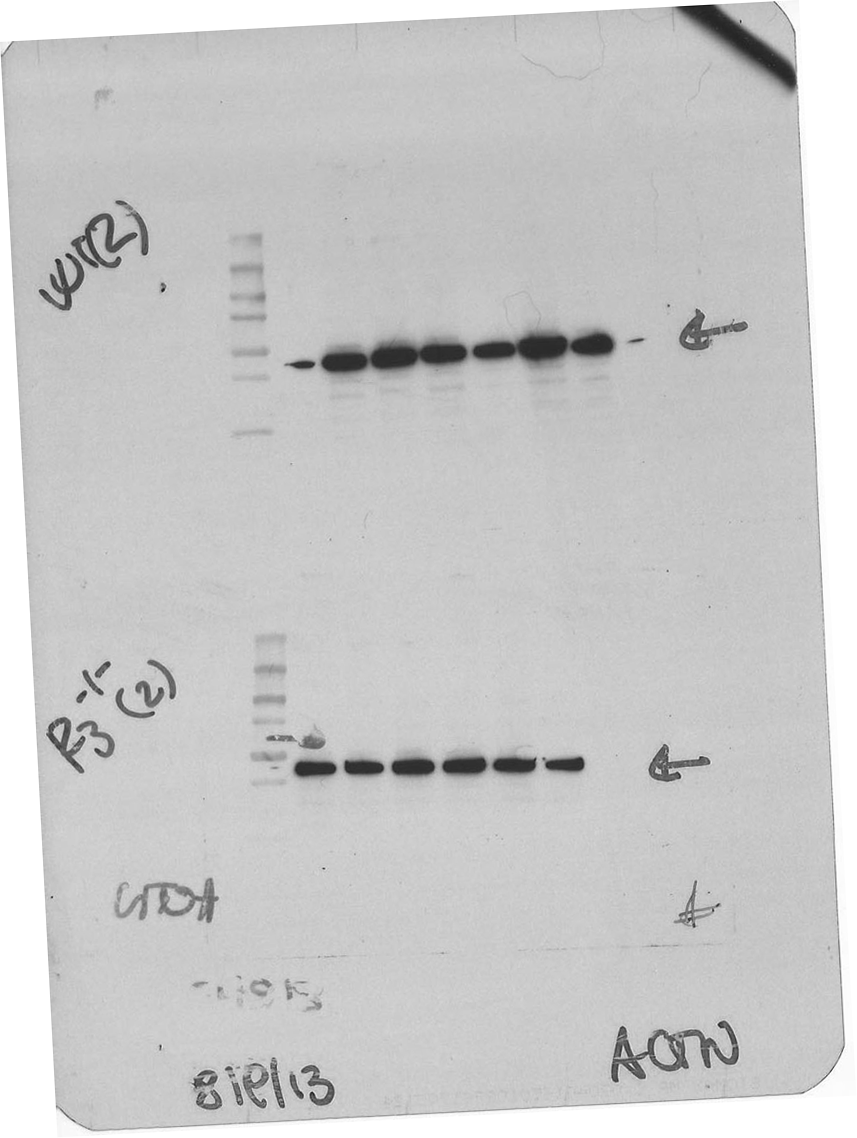

Supplement: Figure 7—source data 2. [file elife-84171-fig7-data2.zip › Figure 7c-source data 3/Figure 7c-Source data 3-actin.tif]

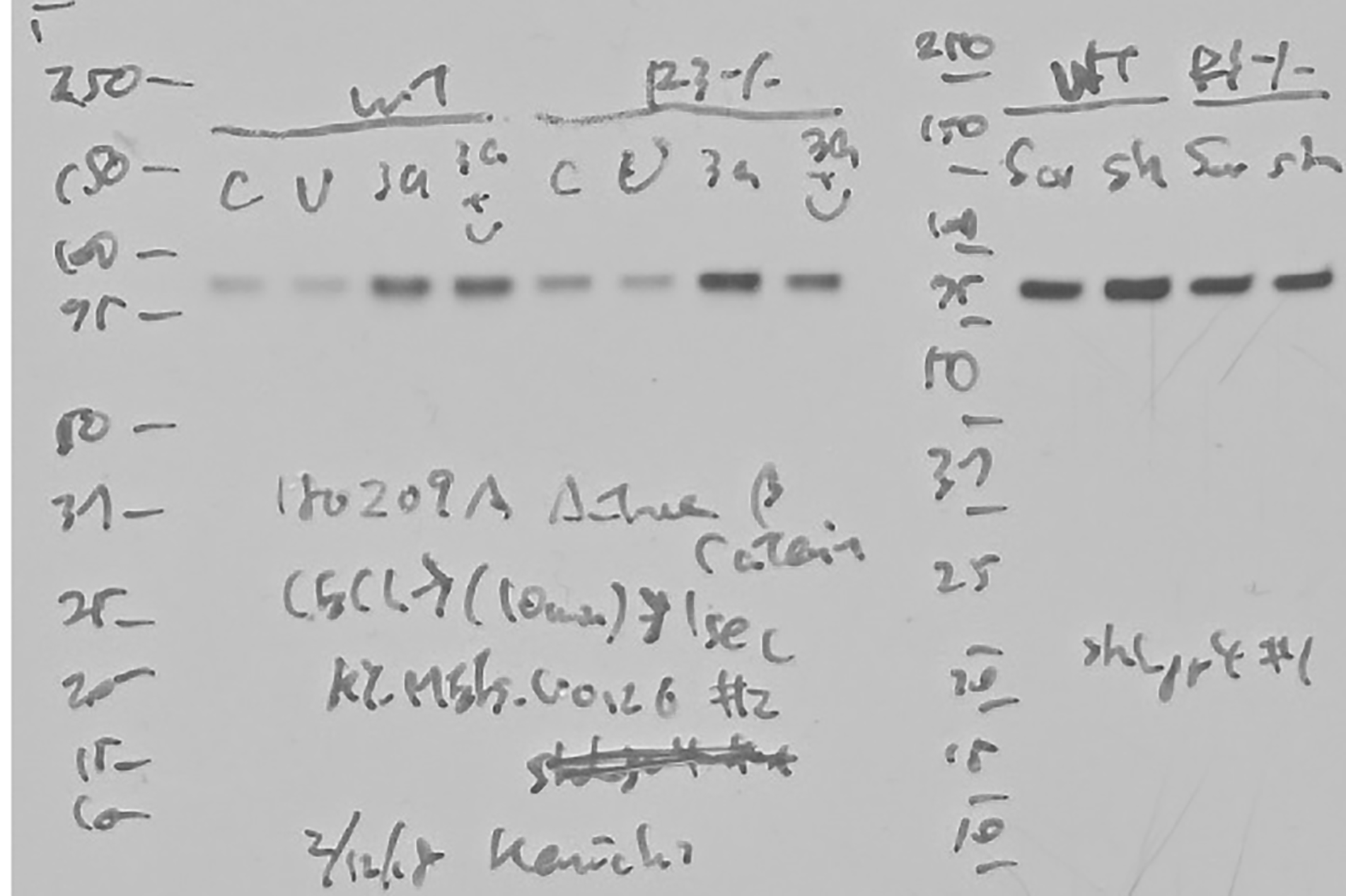

Supplement: Figure 8—source data 1. — Representative image of active β-catenin in WT and Rspo3-/- MEFs treated w/wo w/wo Wnt3a and U0126 (n=3–4). [file elife-84171-fig8-data1.zip › Figure 8a-source data 1/Figure 8a-source data 1 b-catenin.tif]

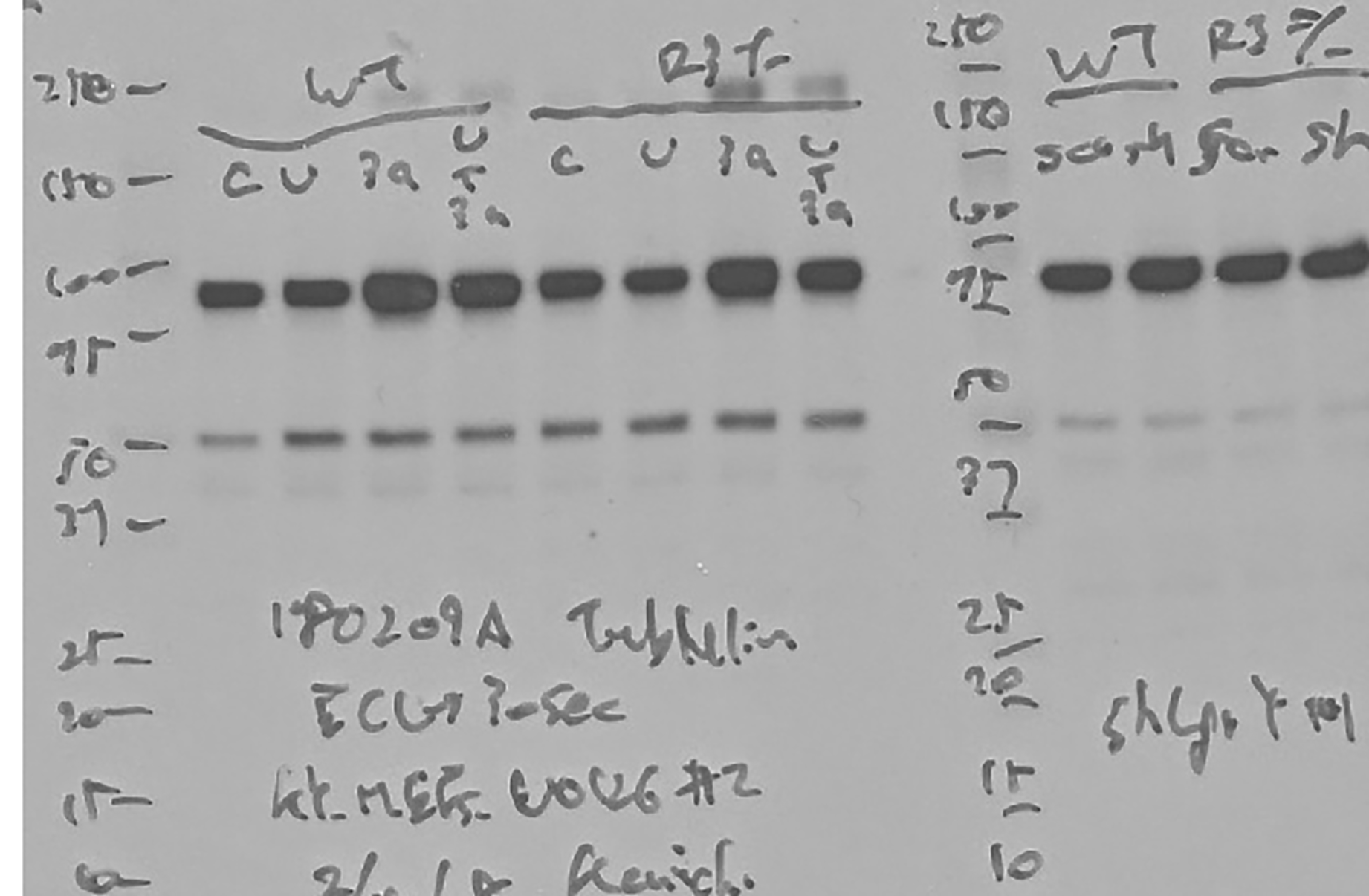

Supplement: Figure 8—source data 2. — Representative image of tubulin levels by western analysis in WT and Rspo3-/- MEFs treated w/wo w/wo Wnt3a and U0126 (n=3–4). [file elife-84171-fig8-data2.zip › Figure 8a-source data 2/Figure 8a-source data 2-tubulin.tif]

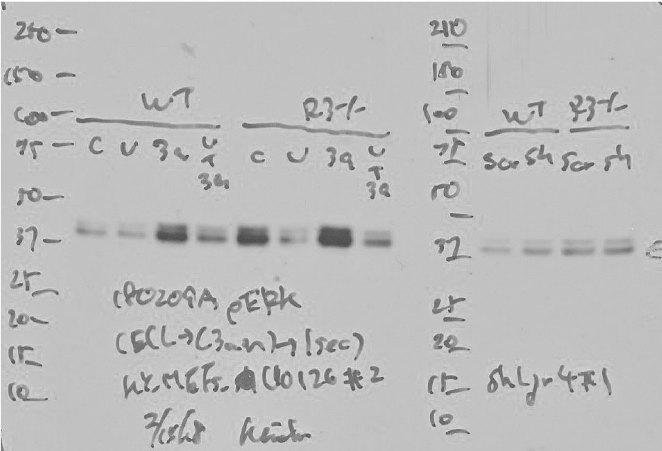

Supplement: Figure 8—source data 3. — Representative image of pERK levels by western analysis in WT and Rspo3-/- MEFs treated w/wo w/wo Wnt3a and U0126 (n=3–4). [file elife-84171-fig8-data3.zip › Figure 8a-source data 3/Figure 8a-source data 3-pERK.tif]

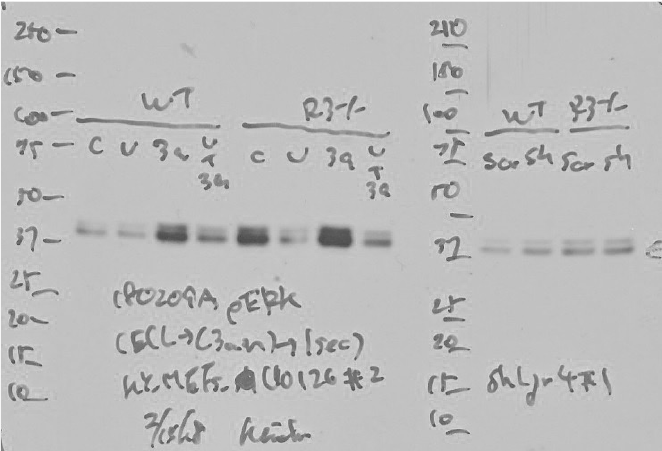


Representative uncropped labelled blot of pErk in *wt* and *Rspo-/-*MEFs treated w/wo w/wo Wnt3a and U0126.

Supplement: Figure 8—source data 3. — Representative image of pERK levels by western analysis in WT and Rspo3-/- MEFs treated w/wo w/wo Wnt3a and U0126 (n=3–4). [file elife-84171-fig8-data3.zip › Figure 8a-source data 3/Figure 8a-source data 3_uncropped label blot.docx]

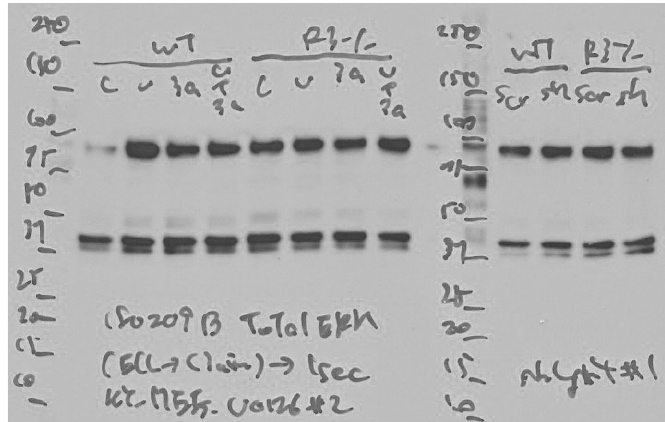

Supplement: Figure 8—source data 4. — Representative image of total ERK levels by western analysis in WT and Rspo3-/- MEFs treated w/wo w/wo Wnt3a and U0126 (n=3–4). [file elife-84171-fig8-data4.zip › figure 8a-source data 4/Figure 8a-source data 4-total ERK.tif]

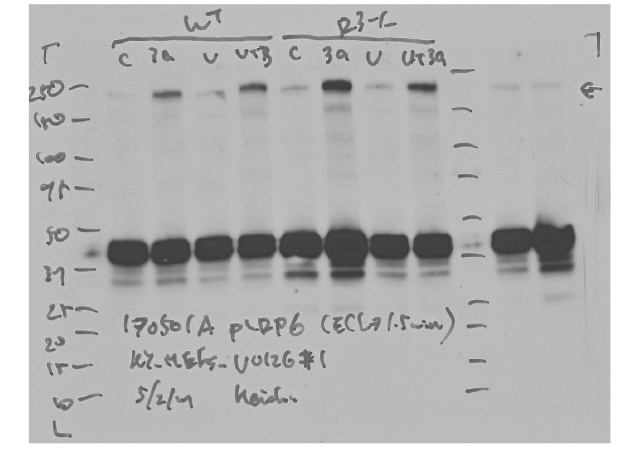


Representative uncropped labelled blot of pLrp6 n *wt* and *Rspo-/-*MEFs treated w/wo w/wo Wnt3a and U0126.

Supplement: Figure 8—source data 5. — Representative image of pLrp6 levels by western analysis in WT and Rspo3-/- MEFs treated w/wo w/wo Wnt3a and U0126 (n=3–4). [file elife-84171-fig8-data5.zip › figure 8a-source data 5/Figure 8a-source data 5-uncropped label blot.docx]

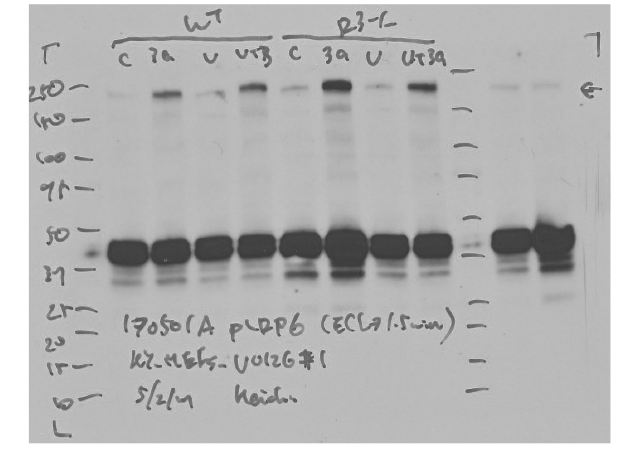

Supplement: Figure 8—source data 5. — Representative image of pLrp6 levels by western analysis in WT and Rspo3-/- MEFs treated w/wo w/wo Wnt3a and U0126 (n=3–4). [file elife-84171-fig8-data5.zip › figure 8a-source data 5/Figure 8a-source data 5-pLRP6.tif]

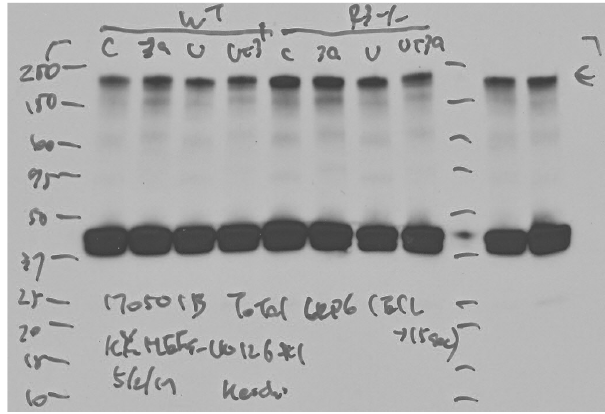

Supplement: Figure 8—source data 6. — Representative image of total Lrp6 levels by western analysis in WT and Rspo3-/- MEFs treated w/wo w/wo Wnt3a and U0126 (n=3–4). [file elife-84171-fig8-data6.zip › figure 8a-source data 6/Figure 8a-source data 6-LRP6.tif]

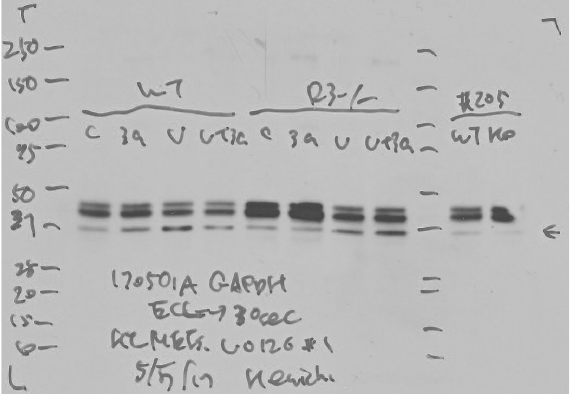


Representative uncropped labelled blot of Gapdh n *wt* and *Rspo-/-*MEFs treated w/wo w/wo Wnt3a and U0126.

Supplement: Figure 8—source data 7. — Representative image of Gapdh levels by western analysis in WT and Rspo3-/- MEFs treated w/wo w/wo Wnt3a and U0126 (n=3–4). [file elife-84171-fig8-data7.zip › Figure 8a-source data 7/Figure 8a-source data 7-uncropped label blot.docx]

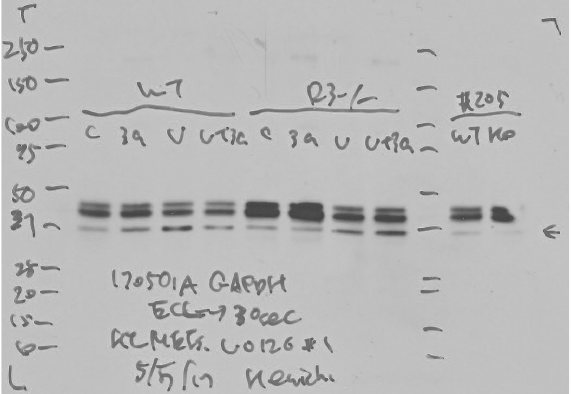

Supplement: Figure 8—source data 7. — Representative image of Gapdh levels by western analysis in WT and Rspo3-/- MEFs treated w/wo w/wo Wnt3a and U0126 (n=3–4). [file elife-84171-fig8-data7.zip › Figure 8a-source data 7/Figure 8a-sourced data 7-GAPDH.tif]
