# Supplementary material for: R-spondin 3 deletion induces Erk phosphorylation to enhance Wnt signaling and promote bone formation in the appendicular skeleton
Source: eLife. 2022 Nov 2;11:e84171. doi: 10.7554/eLife.84171 (PMC9681208; doi:10.7554/eLife.84171)
Supplement: Figure 7—source data 1. — Representative image of active β-catenin and pLrp6 by Western analysis in WT and Rspo3-/- MEFs treated w/wo Wnt3a and increasing doses of Dkk1 (n=3). [file elife-84171-fig7-data1.zip › Figure 7c-source data 1-2/Figure 7c-source data 1-2_uncropped labelled blot.docx]

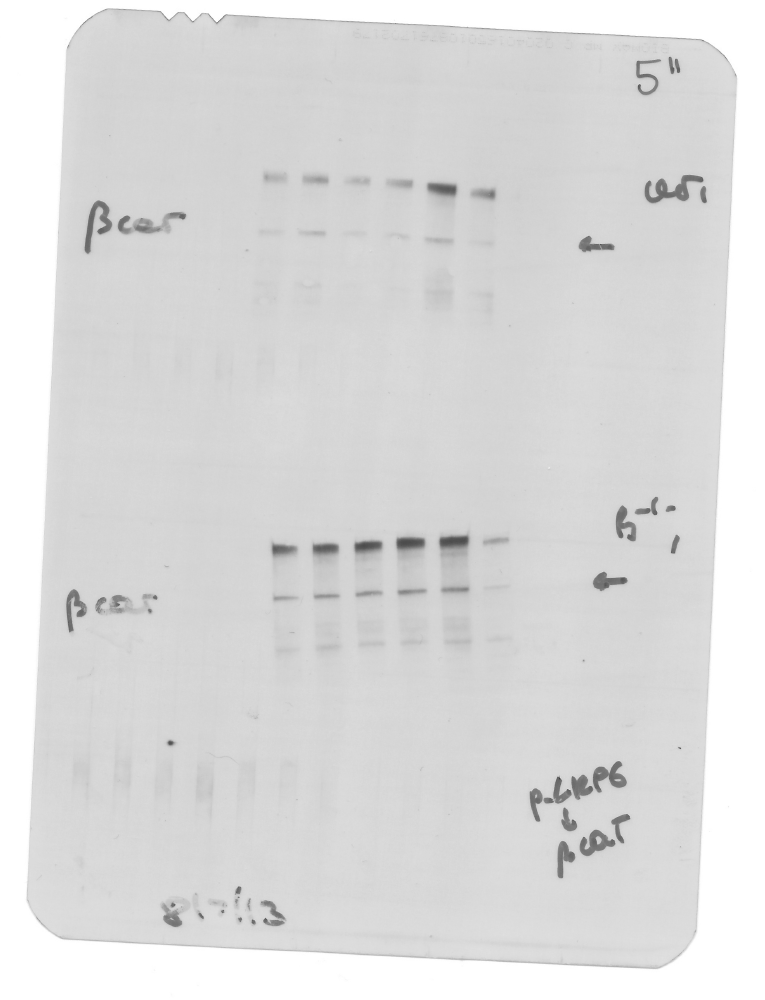


wnt3a

Dkk1

vehicle

wnt3a

Dkk1

vehicle

pLrp6

pLrp6

b-catenin

b-catenin

wt

Rspo3-/-

Representative uncropped labelled blot of pLrp6 and b-catenin in *wt* and *Rspo^-/-^* MEFs treated w/wo Wnt3a and increasing doses of Dkk1.
