# Supplementary material for: R-spondin 3 deletion induces Erk phosphorylation to enhance Wnt signaling and promote bone formation in the appendicular skeleton
Source: eLife. 2022 Nov 2;11:e84171. doi: 10.7554/eLife.84171 (PMC9681208; doi:10.7554/eLife.84171)
Supplement: Figure 7—source data 2. [file elife-84171-fig7-data2.zip › Figure 7c-source data 3/Figure 7c-source data 3_uncropped labelled blot.docx]

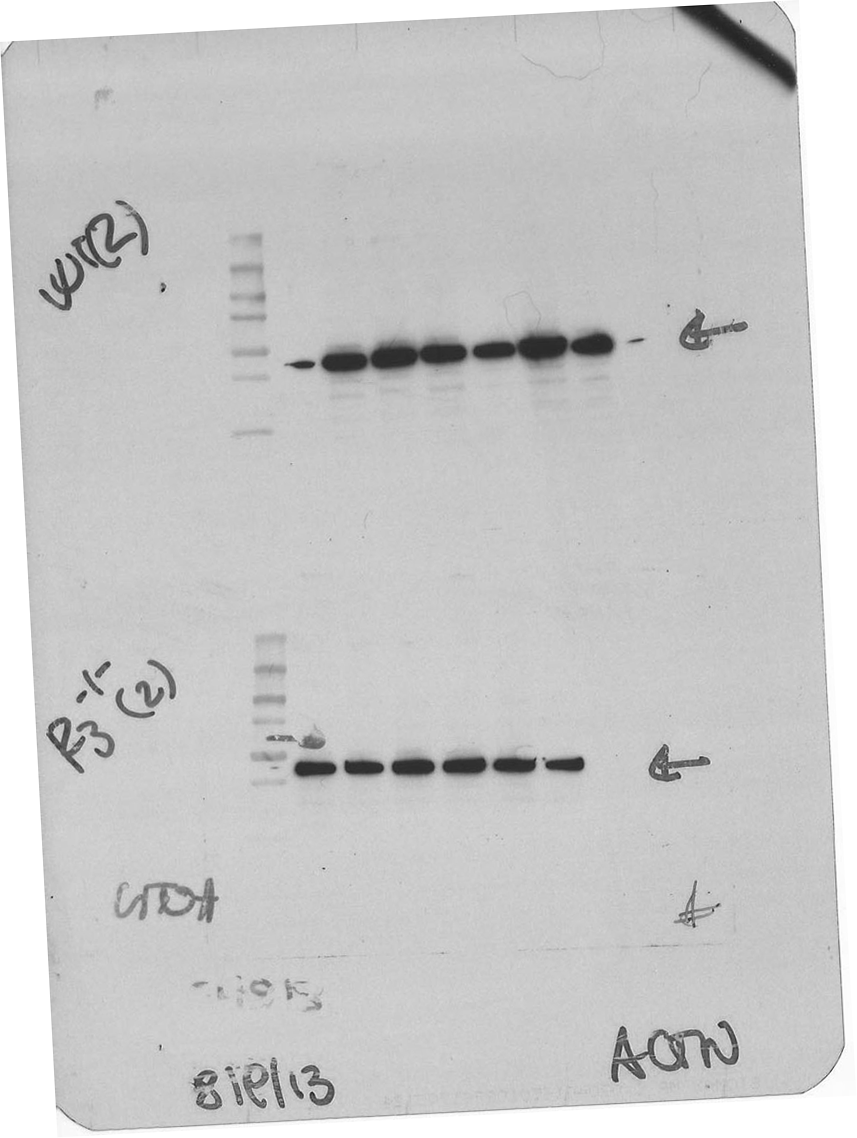


Dkk1

wnt3a

vehicle

Dkk1

wnt3a

vehicle

wt

Rspo3-/-

Representative uncropped labelled blot of actin in *wt* and *Rspo^-/-^* MEFs treated w/wo Wnt3a and increasing doses of Dkk1.
