# Supplementary material for: R-spondin 3 deletion induces Erk phosphorylation to enhance Wnt signaling and promote bone formation in the appendicular skeleton
Source: eLife. 2022 Nov 2;11:e84171. doi: 10.7554/eLife.84171 (PMC9681208; doi:10.7554/eLife.84171)
Supplement: Figure 8—source data 1. — Representative image of active β-catenin in WT and Rspo3-/- MEFs treated w/wo w/wo Wnt3a and U0126 (n=3–4). [file elife-84171-fig8-data1.zip › Figure 8a-source data 1/Figure 8a-source data 1_uncropped label blot.docx]

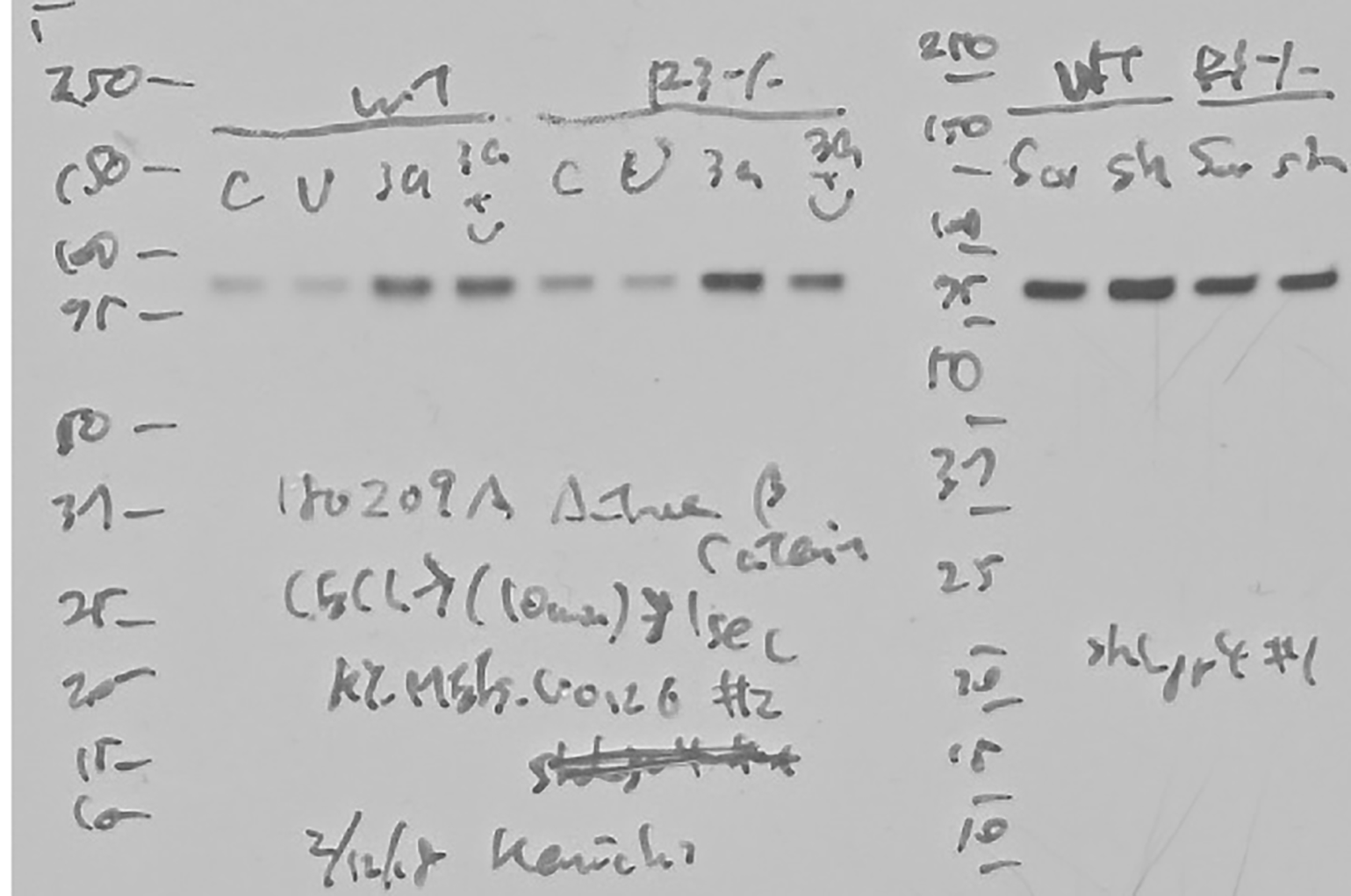


Representative uncropped labelled blot of b-catenin in *wt* and *Rspo^-/-^* MEFs treated w/wo w/wo Wnt3a and U0126.
