# Supplementary material for: R-spondin 3 deletion induces Erk phosphorylation to enhance Wnt signaling and promote bone formation in the appendicular skeleton
Source: eLife. 2022 Nov 2;11:e84171. doi: 10.7554/eLife.84171 (PMC9681208; doi:10.7554/eLife.84171)
Supplement: Supplementary file 1. [file elife-84171-supp1.docx]

**Table S1.** Histomorphometric analysis of WT *and Rspo3^+/-^* males.

| Parameters | 6 wk | | 12 wk | | 18 wk | | Two Way ANOVA | | |
| --- | --- | --- | --- | --- | --- | --- | --- | --- | --- |
|  | **WT**  **(n=8)** | ***Rspo3^+/-^***  **(n=7)** | **WT**  **(n=8)** | ***Rspo3^+/-^***  **(n=9)** | **WT**  **(n=8)** | ***Rspo3^+/-^***  **(n=6)** | **Genotype** | **Age** | **Interaction** |
| BV/TV (%) | 10.9±1.50 | 10.4±1.41 | 9.18±1.10 | 14.3±1.23** | 8.18±0.56 | 10.7±1.19 | 0.0204 | NS | NS |
| Tb.Th (μm) | 30.9±1.94 | 29.0±1.74 | 34.7±2.01 | 41.7±1.96* | 34.2±1.17 | 39.4±2.02* | 0.0248 | <0.0001 | 0.046 |
| Tb.N (/mm) | 3.45±0.30 | 3.51±0.30 | 2.61±0.19 | 3.41±0.22* | 2.38±0.12 | 2.68±0.22 | 0.0476 | 0.0014 | NS |
| Tb.Sp (μm) | 273±27.1 | 270±29.2 | 365±32.1 | 265±24.8** | 394±19.4 | 348±30.9 | 0,033 | 0.005 | NS |
| MAR (μm/day) | 1.58±0.14 | 2.01±0.13** | 1.23±0.07 | 1.54±0.07* | 1.07±0.05 | 1.21±0.06 | 0.001 | <0.0001 | NS |
| MS/BS (%) | 23.3±1.17 | 20.7±2.6 | 45.3±3.60 | 43.0±2.65 | 26.1±2.58 | 29.4±2.30 | NS | <0.0001 | NS |
| BFR/BS  (μm^3^/ μm^2^/year) | 136.3±16.5 | 151±20.4 | 201±13.9 | 242±22.1 | 103±14.0 | 130±12.5** | 0.029 | <0.0001 | NS |
| N.Ob/B.Pm (/mm) | 4.55±0.37 | 5.17±0.21 | 4.94±0.61 | 7.39±0.97** | 4.05±0.48 | 6.93±0.62** | 0.0006 | NS | NS |
| Ob.S/B.Pm (%) | 7.00±0.57 | 8.80±0.38 | 6.39±0.92 | 10.4±1.58** | 6.30±0.74 | 11.2±0.93** | 0.0001 | NS | NS |
| OS/BS (%) | 2.87±0.38 | 3.18±0.29 | 4.62±1.10 | 6.81±1.02* | 4.16±0.62 | 6.42±0.54 | 0.0186 | 0.0033 | NS |
| O.Th (μm) | 2.91±0.23 | 3.61±0.47 | 3.09±0.22 | 4.04±0.23* | 3.65±0.40 | 4.44±0.29 | 0.0033 | NS | NS |
| N.Oc/B.Pm (/mm) | 0.95±0.08 | 0.95±0.15 | 2.29±0.11 | 2.12±0.26 | 1.33±0.07 | 1.39±0.15 | NS | <0.0001 | NS |
| Oc.S/B.Pm (%) | 2.95±0.40 | 2.63±0.32 | 5.87±0.24 | 5.84±0.56 | 3.83±0.25 | 4.17±0.42 | NS | <0.0001 | NS |
| ES/BS (%) | 4.27±0.52 | 4.10±0.53 | 1.61±0.33 | 1.65±0.26 | 4.38±0.27 | 4.93±0.35 | NS | <0.0001 | NS |

Data are expressed as Mean±SEM. Two Way ANOVA followed by Fisher’s LSD post-hoc test

*=p<0.05, **=p<0.005 compared to age-matched WT males.
