# Supplementary material for: R-spondin 3 deletion induces Erk phosphorylation to enhance Wnt signaling and promote bone formation in the appendicular skeleton
Source: eLife. 2022 Nov 2;11:e84171. doi: 10.7554/eLife.84171 (PMC9681208; doi:10.7554/eLife.84171)
Supplement: Supplementary file 2. [file elife-84171-supp2.docx]

**Table S2.** Histomorphometric analysis of the tibia midshaft in 12 wk-old WT and *Rspo3^+/-^* females.

| Parameters | 6 wk | | 12 wk | | 18 wk | | Two Way ANOVA | | |
| --- | --- | --- | --- | --- | --- | --- | --- | --- | --- |
|  | **WT**  **(n=6)** | ***Rspo3^+/-^***  **(n=6)** | **WT**  **(n=4)** | ***Rspo3^+/-^***  **(n=4)** | **WT**  **(n=8)** | ***Rspo3^+/-^***  **(n=6)** | **Genotype** | **Age** | **Interaction** |
| Ct.Ar[mm^2^] | 0.32±0.01 | 0.32±0.007 | 0.369±0.02 | 0.404±0.02 | 0.42±0.014 | 0.42±0.015 | NS | <0.0001 | NS |
| Ma.Ar[mm^2^] | 0.49±0.02 | 0.55±0.02 | 0.614±0.05 | 0.684±0.05 | 0.71±0.01 | 0.6±0.04 | NS | <0.0001 | 0.01 |
| Tt.Ar[mm^2^] | 0.81±0.02 | 0.87±0.03 | 0.983±0.05 | 1.052±0.07 | 1.12±0.02 | 1.0±0.03 | NS | <0.0001 | 0.02 |
| Ct.BV/TV (%) | 40±1.7 | 36.9±0.9 | 37.85±2.74 | 38.53±0.6 | 37.4±0.1 | 42.5±2.2 | NS | NS | 0.046 |
| Ct.Th (mm) | 148.1±5.9 | 147.8±5 | 199±11 | 212±10 | 165±9 | 178±8.4 | NS | <0.0001 | NS |

Data are expressed as Mean±SEM. Two Way ANOVA followed by Fisher’s LSD post-hoc test
