# Supplementary material for: R-spondin 3 deletion induces Erk phosphorylation to enhance Wnt signaling and promote bone formation in the appendicular skeleton
Source: eLife. 2022 Nov 2;11:e84171. doi: 10.7554/eLife.84171 (PMC9681208; doi:10.7554/eLife.84171)
Supplement: Supplementary file 3. [file elife-84171-supp3.docx]

**Table S3.** Histomorphometric analysis of the tibia midshaft in 12 wk-old WT and *Rspo3^+/-^* males.

| Parameters | 6 wk | | 12 wk | | 18 wk | | Two Way ANOVA | | |
| --- | --- | --- | --- | --- | --- | --- | --- | --- | --- |
|  | **WT**  **(n=6)** | ***Rspo3^+/-^***  **(n=6)** | **WT**  **(n=5)** | ***Rspo3^+/-^***  **(n=5)** | **WT**  **(n=8)** | ***Rspo3^+/-^***  **(n=6)** | **Genotype** | **Age** | **Interaction** |
| Ct.Ar[mm^2^] | 0.35±0.02 | 0.34±0.01 | 0.964±0.08 | 0.85±0.085 | 0.42±0.02 | 0.4±0.017 | NS | <0.0001 | NS |
| Ma.Ar[mm^2^] | 0.50±0.03 | 0.49±0.0.02 | 1.181±0.08 | 1.087±0.07 | 0.74±0.04 | 0.68±0.05 | NS | <0.0001 | NS |
| Tt.Ar[mm^2^] | 0.85±0.02 | 0.86±0.02 | 2.145±0.15 | 2.112±0.142 | 1.16±0.05 | 1.07±0.06 | NS | <0.0001 | NS |
| Ct.BV/TV (%) | 41.2±2.47 | 40.9±0.77 | 44.85±1.84 | 48.4±1.52 | 36.4±0.1 | 36.8±1.41 | NS | <0.0001 | NS |
| Ct.Th (mm) | 157.4±15 | 147±2.7 | 203±12 | 214±13 | 180±12 | 190±14 | NS | 0.0007 | NS |

Data are expressed as Mean±SEM. Two Way ANOVA followed by Fisher’s LSD post-hoc test
