# Supplementary material for: R-spondin 3 deletion induces Erk phosphorylation to enhance Wnt signaling and promote bone formation in the appendicular skeleton
Source: eLife. 2022 Nov 2;11:e84171. doi: 10.7554/eLife.84171 (PMC9681208; doi:10.7554/eLife.84171)
Supplement: Supplementary file 4. [file elife-84171-supp4.docx]

**Table S4.** Histomorphometric analysis of 12 wk-old WT and *Rspo3^+/-^* vertebrae.

| Parameters | WT  females (n=6) | *Rspo3^+/-^*  females  (n=7) | WT  males  (n=7) | *Rspo3^+/-^*  males  (n=7) |
| --- | --- | --- | --- | --- |
| BV/TV (%) | 11.6±0.59 | 11.2±1.3 | 16.44±1.27 | 15.7±0.82 |
| Tb.Th (mm) | 36.8±1.56 | 34.8±1.3 | 37.75±1.81 | 37.2±0.93 |
| Tb.N (/mm) | 3.15±0.08 | 3.15±0.21 | 4.32±0.15 | 4.21±0.13 |
| Tb.Sp (μm) | 281.1±8.4 | 293.3±24.8 | 195.2±8.8 | 201.9±8.9 |
| MAR (μm/day) | 1.47±0.07 | 1.46±0.08 | 1.05±0.01 | 1.08±0.04 |
| MS/BS (%) | 35.9±2.31 | 39±2.25 | 28.8±2.8 | 29.5±3.11 |
| BFR/BS(μm^3^/μm^2^/year) | 526±33 | 579.1±56.1 | 303±30 | 319±38 |
| Ob.S/B.Pm (%) | 30.07±3.06 | 35.1±2.45 | 17.51±1.93 | 15.9±1.47 |
| N.Ob/B.Pm (/mm) | 23.51±2.44 | 26.85±1.76 | 13.48±1.47 | 12.51±1.25 |
| OS/BS (%) | 18.16±2.9 | 22.02±2.77 | 10.78±2.12 | 8.83±0.89 |
| O.Th (μm) | 2.71±0.09 | 2.53±0.13 | 2.44±0.19 | 2.84±0.12 |
| N.Oc/B.Pm (/mm) | 7.23±0.31 | 6.26±0.3 | 4.96±0.18 | 4.52±0.24 |
| Oc.S/B.Pm (%) | 16.5±0.72 | 13.4±0.89 | 10.12±0.47 | 9.7±0.57 |

Data are expressed as Mean±SEM.
