# Supplementary material for: R-spondin 3 deletion induces Erk phosphorylation to enhance Wnt signaling and promote bone formation in the appendicular skeleton
Source: eLife. 2022 Nov 2;11:e84171. doi: 10.7554/eLife.84171 (PMC9681208; doi:10.7554/eLife.84171)
Supplement: Supplementary file 5. [file elife-84171-supp5.docx]

**Table S5.** Histomorphometric analysis of 8 wk-old *Rspo3^fl^* and Rspo3-OB-cKO mice.

| Parameters | Male | | Female | |
| --- | --- | --- | --- | --- |
|  | ***Rspo3^fl^***  **(n=10)** | **Rspo3-OB-cKO**  **(n=10)** | ***Rspo3^fl^***  **(n=7)** | **Rspo3-OB-cKO**  **(n=9)** |
| BV/TV (%) | 6.18±0.37 | 10.5±0.74**** | 4.59±0.52 | 6.61±0.52* |
| Tb.Th (μm) | 21.0.4±0.61 | 25.3±1.16** | 19.8±0.05 | 21±0.6 |
| Tb.N (/mm) | 2.94±0.18 | 4.15±0.18** | 2.32±0.27 | 3.11±0.17* |
| Tb.Sp (μm) | 333.4±22 | 220±10.5** | 451±56.5 | 311±21* |
| MAR (μm/day) | 1.03±0.05 | 1.7±0.07**** | 1.32±0.11 | 1.73±0.05** |
| MS/BS (%) | 31.2±1.16 | 40.4±2.02** | 35.2±3.24 | 39.0±1.38 |
| BFR/BS (μm^3^/ μm^2^/year) | 118.3±9.08 | 251±17.3**** | 167±16.4 | 246±11.66** |
| N.Ob/B.Pm (/mm) | 8.71±1.01 | 15.8±2.16* | 8.71±1.51 | 13±0.1.06* |
| Ob.S/B.Pm (%) | 7.50±0.94 | 14.2±2.44* | 8.33±1.59 | 12.7±1.05* |
| OS/BS (%) | 4.42±0.73 | 10.2±1.88** | 8.33±1.29 | 11.9±1.52 |
| O.Th (μm) | 1.99±0.18 | 2.72±0.20* | 1.99±0.19 | 2.29±0.10 |
| N.Oc/B.Pm (/mm) | 3.45±0.27 | 2.8±0.27 | 3.83±0.31 | 3.22±0.20 |
| Oc.S/B.Pm (%) | 6.54±0.41 | 5.46±0.39 | 7.83±0.46 | 6.39±0.43 |
| ES/BS (%) | 1.67±0.23 | 1.53±0.41 | 2.15±0.31 | 1.92±0.32 |

Data are expressed as Mean±SEM. *=p<0.05, **=p<0.01 by unpaired Student’s T-Test compared to age-matched *Rspo3^fl^* mice.
