# Supplementary material for: R-spondin 3 deletion induces Erk phosphorylation to enhance Wnt signaling and promote bone formation in the appendicular skeleton
Source: eLife. 2022 Nov 2;11:e84171. doi: 10.7554/eLife.84171 (PMC9681208; doi:10.7554/eLife.84171)
Supplement: Supplementary file 6. [file elife-84171-supp6.docx]

**TableS6.** Histomorphometric analysis of 8 wk-old *Rspo3^fl^* and *Rspo3-OB-cKO* vertebrae.

| Parameters | Male | | Female | |
| --- | --- | --- | --- | --- |
|  | ***Rspo3^fl^***  **(n=10)** | ***Rspo3-OB-cKO***  **(n=10)** | ***Rspo3^fl^***  **(n=7)** | ***Rspo3-OB-cKO***  **(n=9)** |
| BV/TV (%) | 19.8±4.28 | 14.5±1.6** | 14.2±0.72 | 15.28±0.71 |
| Tb.Th (mm) | 39.4±1.6 | 36.4±1 | 34.7±0.85 | 34.6±0.84 |
| Tb.N (/mm) | 4.97±0.17 | 3.99±0.25** | 4.08±0.12 | 4.4±0.12 |
| Tb.Sp (μm) | 164±8.7 | 223±21.7* | 212.3±7.5 | 195±6.7 |
| MAR (μm/day) | 1.54±0.04 | 1.57±0.06 | 2.29±0.07 | 2.24±0.07 |
| MS/BS (%) | 31.01±1.3 | 34±2.34 | 40.5±1.17 | 43.13±0.82 |
| BFR/BS (μm^3^/ μm^2^/year) | 478±25 | 543±52 | 933±51 | 968±73 |
| N.Ob/B.Pm (/mm) | 4.65±0.66 | 8.8±2.3 | 15.9±3.53 | 14.3±1.76 |
| Ob.S/B.Pm (%) | 6.3±0.9 | 11.63±2.5 | 21.8±4.76 | 20.3±2.5 |
| OS/BS (%) | 4.42±0.73 | 10.2±1.8 | 8.33±1.29 | 11.9±1.52 |
| O.Th (μm) | 2.1±0.14 | 2.3±0.22 | 2.25±0.14 | 2.05±0.06 |
| N.Oc/B.Pm (/mm) | 7.26±0.3 | 9.94±0.31**** | 11.5±0.6 | 12.1±0.6 |
| Oc.S/B.Pm (%) | 6.54±0.41 | 5.46±0.39 | 7.83±0.46 | 6.39±0.43 |

Data are expressed as Mean±SEM. *=p<0.05, **=p<0.01, ****=p<0.001 by unpaired Student’s T-Test

compared to sex-matched *Rspo3^fl^* mice.
