# Supplementary material for: R-spondin 3 deletion induces Erk phosphorylation to enhance Wnt signaling and promote bone formation in the appendicular skeleton
Source: eLife. 2022 Nov 2;11:e84171. doi: 10.7554/eLife.84171 (PMC9681208; doi:10.7554/eLife.84171)
Supplement: Supplementary file 7. [file elife-84171-supp7.docx]

**Table S7.** Histomorphometric analysis of *Control,* *Rspo3^+/-,^Dkk-Tg* and *Rspo3^+/-^;Dkk1-Tg* female mice at 6 wk of age.

| Parameter | *Control*  (n=5) | *Rspo3^+/-^*  (n=5) | *Dkk1-Tg*  (n=5) | *Rspo3^+/-^;Dkk1;Tg*  (n=6) | Two Way ANOVA | | |
| --- | --- | --- | --- | --- | --- | --- | --- |
|  |  |  |  |  | ***Rspo3*** | ***Dkk1*** | **Interaction** |
| BV/TV (%) | 5.02±0.57 | 7.83±0.75^ac^ | 2.67±0.44^ab^ | 4.64±0.5^bc^ | 0.0008 | 0.0002 | NS |
| Tb.Th (μm) | 29.0±1.65 | 32.2±2.07 | 28.8±2.34 | 32.6±2.47 | NS | NS | NS |
| Tb.N (/mm) | 1.71±0.11 | 2.4±0.01^ac^ | 0.9±0.1^ab^ | 1.43±0.12^bc^ | <0.0001 | <0.0001 | NS |
| Tb.Sp (μm) | 586.1±30.9 | 390.4±17.9^c^ | 1202±154^ab^ | 701.7±61.1^bc^ | <0.0001 | <0.0001 | 0.0082 |
| MAR (μm/day) | 1.63±0.2 | 2.12±0.27 | 1.12±0.13^b^ | 1.97±0.18^c^ | 0.007 | NS | NS |
| MS/BS (%) | 35.19±2.06 | 37.7±2.37 | 30.8±4.8 | 42.9±2.04^c^ | 0.0194 | NS | NS |
| BFR/BS(μm^3^/μm^2^/year) | 212.8±34 | 296±45.8^c^ | 127.8±27.2^b^ | 307.4±38.3^c^ | 0.0064 | NS | NS |
| Ob.S/B.Pm (%) | 18.75±1.63 | 25.5±1.1^ac^ | 8.9±1.16 ^ab^ | 20.0±2.35^bc^ | <0.0001 | 0.0004 | NS |
| N.Ob/B.Pm (/mm) | 14.6±0.92 | 19.3±0.41^ac^ | 7.2±0.82^ab^ | 15.2±1.65^bc^ | <0.0001 | 0.0001 | NS |
| OS/BS (%) | 13.05±1.53 | 16.9±1.84^c^ | 7.32±1.9^b^ | 13.6±2.6^c^ | 0.0261 | 0.0435 | NS |
| O.Th (μm) | 4.35±0.30 | 4.80±0.32^c^ | 2.68±0.51^ab^ | 4±0.25^c^ | 0.025 | 0.0025 | NS |
| N.Oc/B.Pm (/mm) | 4.22±0.37 | 4.56±0.27^c^ | 6.31±0.46^ab^ | 5.11±0.38^c^ | NS | 0.0064 | NS |
| Oc.S/B.Pm (%) | 12.58±0.6 | 12.77±0.51^c^ | 16.9±1.18^ab^ | 14.1±1.31^c^ | NS | 0.0087 | NS |
| ES/BS (%) | 4.51±0.7 | 4.46±0.47 | 5.14±0.48 | 5.8±0.74 | NS | NS | NS |

Data are expressed as Mean±SEM. Two-Way ANOVA followed by Fisher’s LSD post-hoc test.

a=p<0.05 compared to *control* mice, b= p<0.05 compared to *Rspo3^+/-^* mice, c= p<0.05 compared to *Dkk1-Tg* mice.
